# Stress, anxiety, self-efficacy, and the meanings that physical therapy students attribute to their experience with an objective structured clinical examination

**DOI:** 10.1186/s12909-020-02202-5

**Published:** 2020-09-10

**Authors:** Érica de Matos Reis Ferreira, Rafael Zambelli Pinto, Paula Maria Machado Arantes, Érica Leandro Marciano Vieira, Antônio Lúcio Teixeira, Fabiane Ribeiro Ferreira, Daniela Virgínia Vaz

**Affiliations:** 1 Department of Physical Therapy, Universidade Federal de Minas Gerais (UFMG), Belo Horizonte, MG Brazil; 2 Interdisciplinary Laboratory of Medical Investigation, School of Medicine, Universidade Federal de Minas Gerais (UFMG), Belo Horizonte, MG Brazil

**Keywords:** OSCE, Stress, Anxiety, Self-efficacy

## Abstract

**Background:**

Excessive stress and anxiety can impair learning. The objective structured clinical examination (OSCE) is a valuable tool to assess and promote the acquisition of clinical skills. However, significant OSCE-related stress and anxiety are frequently reported. The aim of this study was to investigate the relationships between physiological stress, self-reported levels of anxiety due to an OSCE, self-efficacy, and the meanings that physical therapy students attribute to their experience with the exam.

**Design:**

Concurrent mixed methods study.

**Methods:**

A total of 32 students took part in this study. All were enrolled in the third semester of a 10-semester Physical Therapy Bachelor Program. Salivary cortisol levels, self-reported anxiety (State-Trait Anxiety Inventory, STAI) were measured before the OSCE. Exam scores and self-efficacy ratings were also recorded. Correlations between variables were tested with the Pearson correlation, with ɑ at 0.05. Semi-structured interviews were used to explore the personal perspectives of students. Thematic analysis was used to investigate emergent themes.

**Results:**

Trait anxiety scores were significantly higher than normative values (*p* < 0.001). A high proportion of students showed high (STAI> 49) state anxiety (37.5%) and trait anxiety (65.6%). Salivary cortisol was not associated anxiety (*p* > 0.05). Neither stress nor anxiety correlated with OSCE scores. A moderate and significant direct correlation was found for self-efficacy scores and OSCE scores (*r* = 0.475, *p* = 0.007). Students reported that confidence had a calming effect and led to better self-perceived performance. They also reported that the OSCE can provide meaningful learning experiences despite being stressful.

**Conclusions:**

A high proportion of our students reported a stable/lingering negative affect. However, neither stress nor anxiety related to OSCE scores. Students’ confidence in their capabilities was correlated with their performance. Their subjective reports suggest that self-confidence may have protected them from the negative effects of stress and anxiety on academic performance.

## Background

Stress and anxiety are highly prevalent among healthcare students worldwide [1] and performance examinations are a major source of such stress and anxiety. Achievement-demanding test situations can lead to fear of poor evaluation that results in negative physiological, emotional, or behavioral responses [2]. Test anxiety is particularly significant for skill demonstration tests, such as the objective structured clinical examination (OSCE) [3–7]. Given the prevalence and importance of OSCEs in healthcare professions education, it is important to understand how OSCE-related stress and anxiety affect students*’* subjective experience and objective performance.

In an OSCE, each student is required to demonstrate specific skills and behaviors in a simulated work environment. An OSCE typically consists of a series of assessment tasks (stations) simulating real-life clinical situations with an actor [4, 8, 9]. Students need to demonstrate their skills within a standard time limit. Performance is assessed by a trained examiner using a predetermined, objective scoring scheme [10].

Most students and raters describe the OSCE as a valuable formative assessment through which they learn which skills are important and need improvement [4, 5, 11–13]. An OSCE can reveal a detailed picture of both student performance and course efficacy, helping clinical tutors to teach more uniformly. The exam may also actually increase students’ drive to study and practice [4, 14, 15]. Despite its potential benefits, many students consider the OSCE more stressful and intimidating than other kinds of tests [4–7]. A within-groups comparison of anxiety just before dental students took four types of assessments (written, OSCE, pre-clinical crown and bridge preparation test, and a non-exam situation) indicated higher anxiety before the OSCE. Across healthcare courses, OSCE-related nervousness, stress, and anxiety are consistently reported [7, 14, 16, 17].

Excessive stress can interfere with the demonstration of actual competence and so interfere with OSCE validity [18, 19]. A high level of test anxiety may also hamper the ability of the student to learn from the test [20]. In addition, examination anxiety and stress may lead to a variety of negative consequences such as low self-esteem, reduced sleep quality, and depression [21].

It is in both the educators’ and the students’ best interests to reduce excessive examination anxiety so that performance is a reliable indicator of actual competence [10] and learning is optimized. Therefore, investigation of OSCE-related stress and anxiety and their subjectively perceived causes, meanings and repercussions for learning is essential for the development of best practices in healthcare education. OSCE-related stress can be quantified at the physiological level. Salivary cortisol on the day of the exam is a reliable quantitative indicator of stress. In a recent study, serum cortisol was positively correlated with the level of stress perception [18]. At the experiential level, test anxiety can be reliably assessed with the State-Trait Anxiety Inventory, an internationally validated questionnaire [22, 23]. Finally, self-efficacy and the meanings that each individual may attribute to the OSCE experience can appreciably change its impact on performance and learning. Qualitative analysis may help educators understand the student’s experience more deeply so that stress triggers can be mitigated and overall OSCE quality can be improved. Thus, the objective of this study was to investigate the relationships between physiological stress, self-reported levels of OSCE-related anxiety, self-efficacy, and the meanings that students attribute to their experience with the exam.

## Methods

### Study design and settings

This concurrent mixed methods study included quantitative and qualitative methods. Data collection took place at Universidade Federal de Minas Gerais, Brazil, in December 2018.

### Participants

This study used a purposive sample with a total of 32 students (average age 21.1 ± 2.1, 26 females) who were enrolled in the third semester of a 10-semester Physical Therapy (PT) Bachelor Program at a leading Brazilian University and had already participated in one OSCE since the beginning of the PT program. This study was approved by the Research Ethics Committee of Universidade Federal de Minas Gerais. All students gave informed consent prior to participation in the study.

### The objective structured clinical examination (OSCE)

Groups of three students took rounds in three stations (one student per station) planned to assess the following skills: ability to communicate with the patient, ability to establish a therapeutic alliance, and ability to verbalize clinical reasoning and make decisions. In each station, the student had 1 minute to read the case description and the task instructions fixed on the door. Once inside, the student had 6 minutes to interact with an actor playing the role of a patient and finish the clinical task. An examiner (a trained PT enrolled on the Graduate Program) observed and rated the student’s performance according to predefined checklists. Examiners did not interact with the students. OSCE feedback was provided in a group session 2 days after the study was concluded.

### Procedures

#### Salivary cortisol

Thirty minutes before the OSCE began students were informed that they could only provide a saliva sample if they had not eaten anything or brushed their teeth 1 hour prior to the moment of providing saliva samples. Thirty students fit the criteria and saliva was collected around 8:00 am by unstimulated passive drool with a straw kept inside the mouth for 180 s and then put into a 4 ml polyethylene tube (Salivette®, Sarstedt, Germany). The samples were stored in the tubes in a − 80 °C freezer. On the day of the analysis, samples were thawed and centrifuged at 3000 rpm. Cortisol concentration was quantified using an immuno-assay kit (Parameter Cortisol Assay). A 96-well ELISA reader was used for analysis (RnDSystems Parameter Cortisol Assay).

#### Subjective perception of anxiety

Twenty minutes before the OSCE, all eligible students completed the Brazilian short version of the State-Trait Anxiety Inventory – STAI [23], an internationally validated questionnaire for levels of test anxiety [24] with good internal consistency and test-retest reliability [25]. The scale measures two components of anxiety: state anxiety (STAI-S), which refers to transitory feelings of anxiety or tension that can vary in intensity over time; and trait anxiety (STAI-T), which refers to a relatively stable disposition to respond to stress and perceived threats with anxiety in a wide range of situations [26].

The Brazilian short version [23] has six statements for each anxiety component (e.g., “I am tense”). Responses indicate the level of agreement with each statement (1 = not at all, 2 = somewhat; 3 = moderately so; 4 = very much so). The scores for state and trait anxiety range from 6 to 24, with higher scores indicating higher levels of anxiety. Prorated scores were obtained by multiplying the total score of each scale to 20/6 [24] to obtain comparability to the original STAI scale scores (ranging from 20 to 80).

#### Meanings of the OSCE experience

After the OSCE, all students were personally invited by an interviewer, in their groups of three, to talk about their perception of the experience. All students agreed to complete one face-to-face interview in an office where no one else was present besides the other two participants and the interviewer. The interviewer was a female Ph.D. Professor at the PT Department with several years of experience and publications using qualitative research methods. The students had taken a course with her the year before, but she was not involved in the OSCE. She explained that the objective of the research was to understand the meanings that PT students attributed to their experience with the exam. She also explained that, as the course coordinator, she intended to use the interview results to improve the next versions of the OSCE. The semi-structured interview was based on a guide covering the following topics: the students’ feelings of anxiety, their opinions about the main challenges of the exam, their thoughts about whether and what they had learned from the experience, and their overall satisfaction level. The interview guide had been tested in a previous OSCE. Each interview took around 7 minutes. The interviewer took notes for all the 11 interviews. The audio was electronically recorded (SONY® recorder) and transcribed for thematic analysis. Transcripts were returned to students and no corrections were necessary. The software Atlas ti version 7.0 was used to group themes and citations.

#### Self-efficacy

After the students completed the OSCE and before feedback about their performance on the exam was given, they answered a self-efficacy questionnaire with 16 statements, each referring to the level of confidence about a particular skill that was tested in the OSCE (Table 1), for example, “I feel confident in my ability to stimulate the patient to participate in decision making.” Items were scored on a Likert scale varying from 1 (completely disagree) to 5 (completely agree), Total scores varied from 16 to 80. Higher scores indicated higher self-efficacy.

**Table 1:** Self-efficacy questionnaire

I feel confident in my ability to…	
1	… treat the patient with kindness and attention
2	… establish a calm and empathic connection with the patient
3	… communicate with simple and accessible language
4	… organize the interview and procedures based on the interests of the patient
5	… investigate the patient’s functional complaints and their circumstances
6	… simulate functional tasks and observe the patient’s performance
7	… decide what to assess first in the physical exam, based on the interview and the clinical status
8	… write results of my assessment in a form
9	… organize written information according to the ICF’s levels of Body functions & structures, Activities and Participation
10	… organize written information according to the ICF’s levels of Personal and Environmental Factors
11	… define therapeutic objectives without confusing them with treatment procedures
12	… negotiate therapeutic objectives with the patient
13	… assess the coherence between therapeutic objectives and treatment procedures
14	… assess the coherence between treatment procedures and examination results
15	… encourage the patient to participate in the definition of the treatment plan
16	… select appropriate procedures to train and improve task performance

### Data analysis

For quantitative data, means and standard deviations were used as descriptive statistics. After Kolmogorov-Smirnov and Shapiro-Wilk tests of normality, the correlations between cortisol levels, state anxiety, trait anxiety, self-efficacy, and OSCE scores were tested with the Pearson product correlation, with the level of significance at ɑ = 0.05. The correlation analysis included only data from participants with complete data (i.e., missing data were not inferred; see Table 3). Cortisol levels and STAI scores were compared reference values with the independent *t-*tests. Normative values for STAI-S and STAI-T have been reported elsewhere [23]. Reference cortisol values were obtained from a study that collected salivary cortisol at a similar time (7:30 am) from similar individuals (22 young adults (15 women) aged 23 ± 3 years) [27].

Thematic analysis [28] was conducted by two researchers (a PT Professor experienced with OSCEs and qualitative analysis (FRF) and a graduate student with little OSCE experience (EMRF). No a priori themes were described; they emerged during the analysis process. Nevertheless, the themes were informed by the researchers’ personal experience with OSCEs and their understanding of the literature. The transcripts were read repeatedly for identification of central and repeating ideas. Codes were defined based on these initial readings. The interviews were then coded by both researchers line by line and grouped into themes. The final set of themes was agreed through discussion and consensus (the coding tree can be made available by request). Inductive saturation was verified during the analysis. The two researchers derived an explanatory model of the student experience that outlined the relationships between themes. Quotations illustrating the themes were identified (each group of three students was identified by a trio number). Findings were presented to students in a group session for participant checking. The students did not express any disagreements.

## Results

All 32 students were considered eligible for this study, with 30 eligible for the provision of saliva samples. Five saliva samples had insufficient volume and could not be analyzed. No blood-contaminated samples were identified [29]. Average cortisol levels for the 25 students with analyzable samples (6.3 ± 3.1 ng/ml) were not significantly different (*p* = 0.69) from values found for a comparable sample (6.6 ± 2.0 ng/ml) [27]. STAI-S scores (12.7 ± 2.0) were not significantly different (*p* = 0.77) from normative values (12.6 ± 3.6) [23]. STAI-T scores (15.0 ± 2.5), however, were significantly elevated (*p* < 0.001) compared to the population norm (12.4 ± 3.7) [23]. Table 2 shows the descriptive data for cortisol levels, prorated STAI-S, STAI-T, OSCE, and self-efficacy scores.

**Table 2:** Descriptive statistics for cortisol levels, prorated STAI-S and STAI-T scores, self-efficacy and OSCE scores. Low indicates values 2 SD below and high indicates values 2 SD above the sample mean, except for STAI scores, for which the classification is based on predefined cut points: low < 33, medium = 33 to 49, and high > 49 [21]

	Mean ± SD	Low	Medium	High
Cortisol (ng/ml)	6.3 ± 3.1	1 (4%)	23 (92%)	1(4%)
STAI-S	44.1 ± 16.5	7 (21.8%)	13 (40.6%)	12 (37.5%)
STAI-T	52.3 ± 12.7	2 (6.25%)	9 (28.1%)	21 (65.6%)
Self-efficacy	51.0 ± 9.4	5 (16.1%)	22 (70.9%)	4 (12.9%)
OSCE scores	18.5 ± 2.0	5 (15.6%)	21 (18.7%)	6 (18.7%)

**Fig. 1:**
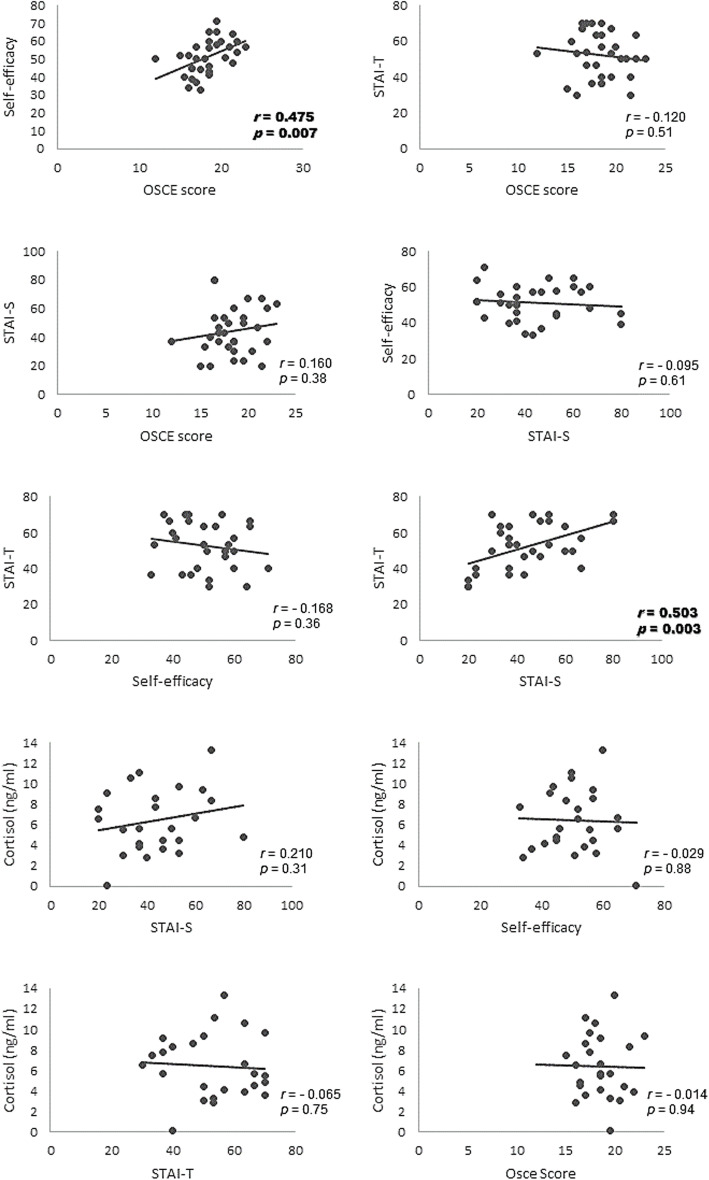
Dispersion diagrams of correlations between dependent variables

Table 3 shows the Pearson *r* correlation coefficients, the number of participants, and the significance values. No significant correlations were found between cortisol levels, STAI scores or OSCE scores (*p* > 0.05). STAI-T and STAI-S scores were significantly correlated with each other (*r* = 0.503, *p* = 0.003). A moderate and significant direct correlation (Fig. 1) was found for self-efficacy scores and OSCE scores (*r* = 0.475, *p* = 0.007).

**Table 3:** Pearson r correlation coefficients, number of data points and significance

	Cortisol	STAI-T	STAI-S	Self-efficacy	OSCE
Cortisol	1				
STAI-T	−.065 (25)	1			
STAI-S	.210 (25)	.503^a^ (32)	1		
Self-efficacy	−.029 (25)	−.168 (31)	−.095 (31)	1	
OSCE	−.014 (25)	−.120 (32)	.160 (32)	.475* (31)	1

*STAI-T* Trait Anxiety score, *STAI-S* State Anxiety score, *OSCE* Objective Structured Clinical Examination score. ^a^Correlation is significant at the 0.01 level

The results of interviews illuminate the relationships between stress, anxiety, OSCE performance, and learning. The following themes emerged during the interviews: 1) previous experience with an OSCE has a calming effect; 2) gaining knowledge and skill has a calming effect; 3) calm leads to better performance; 4) poor interpersonal skills increase anxiety; and 5) perceived gaps between theory and practice increase anxiety. Students reported an important reduction in anxiety compared to the first time they took part in an OSCE, mainly because they were now familiar with the assessment structure:

*"This was less stressful than the first; you know what to expect. It's like your first driving test, the second time you already know how it goes."* (trio 6)

Students felt more confident due to their perception of having acquired the knowledge and skills that were necessary for making appropriate judgments and action choices:

*"I was less stressed because we were technically prepared this time; the classes were very helpful."* (trio 2)

*"There is still a lot to improve, but it was less stressful. We've studied it, I know it. I may not have done things in the best way, but it is something I know."* (trio 8)

Nevertheless, one student reported that being more aware of the necessary skills made her feel greater pressure to apply the acquired knowledge:

*"I felt more confident in the first OSCE compared to this one because in this one I was more worried about what to ask the patient, how to establish good rapport, and how to write it all down."* (trio 11)

In general, however, confidence had a calming effect and led to the perception of better performance. In contrast, the sense of being observed during the exam led to anxiety due to shyness and self-consciousness:

*"It is hard when you know someone is observing and assessing you… I feel unsure."* (trio 9)

*“I was embarrassed, I could not perform the way I wanted.”* (trio 2).

Fear of not being able to apply theoretical knowledge to practical tasks also led to anxiety. However, the experience of these feelings was valued as a source of self-knowledge about personal strengths and weaknesses as well as reflections on how to improve oneself and acquire more confidence:

*"It's about preparation! You can see your difficulties and work on them … It is totally different from practicing with your classmates. The way you interact… in there, you have to be the physical therapist, right?"* (trio 2)

*"Regarding the proper way to interact with a patient, I think it is very pertinent. You know, you can see the skills you need to try and acquire what is missing, what needs to improve and the best way to approach the person."* (trio 7)

Students reported that the experience was meaningful to their education:

*"Because we need to feel anxious about the interaction with other people so that when you get there, you think: I've done this before! It was just a little scene, but it has prepared me for the real moment tomorrow”.* (trio 8)

*"It’s a simulation of clinical practice. It's just like behavior: you learn it by doing it. We can have a thousand lectures on the therapeutic process and how to treat a patient but, if you never do it, you won't know."* (trio 10)

**Fig. 2:**
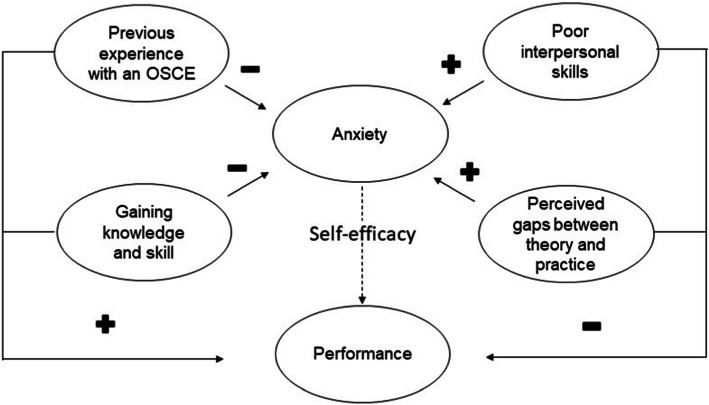
Relationship between themes. Plus and minus signs indicate factors that positively or negatively affect anxiety and performance. The dotted line indicates a moderation effect

The model developed from the thematic analysis of interviews is presented in Fig. 2. It shows that OSCE-related anxiety is influenced by multiple factors and that the relationship between anxiety and performance appears to be moderated by the sense of self-efficacy.

## Discussion

This study investigated how PT students experienced an OSCE exam with respect to physiological levels of cortisol, self-reported levels of anxiety, self-efficacy, and perceived meanings. An understanding of OSCE-related anxiety and its relation to self-efficacy and to the overall subjective interpretation of the experience is essential for the development of best practices in PT education [10].

An interesting first finding in this study is the absence of a relationship between cortisol levels and self-reported anxiety (STAI scores). Cortisol is considered the main biomarker in stress research [30] and is expected to be positively correlated with subjective stress responses, in particular, self-reported anxiety [31]. However, our study found no correlation between cortisol levels and STAI-trait or STAI-state scores preceding the OSCE. Although several studies have found increases in cortisol levels preceding exam situations [32], many studies have found no associations between cortisol concentrations and self-reported anxiety, in line with our study [31, 33].

We surmise two reasons for the lack of association. The first is that STAI-T scores may have reflected significant aspects of depression rather than anxiety [34] in our sample. The second is that self-efficacy might have attenuated state anxiety (captured by STAI-S scores), thus decoupling physiological (cortisol) and psychological (anxiety) stress responses [33, 35].

Our average scores for the short STAI-S were not significantly different from Brazilian normative data. STAI-T scores, in contrast, were significantly elevated in comparison to previously established norms [23]. The distribution of scores among students reveals that a substantial proportion of the students experienced significant anxiety (Table 2). Prorated scores indicate that 37.5% of our students showed high state anxiety and 65.6% showed high trait anxiety (scores above 49 [34, 36, 37].

A high prevalence of anxiety among health students has been reported worldwide [1]. A recent study has shown that 30.4% of 1350 students in 22 Brazilian medical schools had high state and trait anxiety [37]. Our indices are higher and cause concern, especially with respect to trait anxiety. Whereas the state anxiety results indicate that an OSCE event is a significant temporary stressor for some students, trait anxiety, in contrast, points to anxiety that is not circumstantial but stable over time. In addition, there is some evidence that the STAI-T appears to assess depression as well as anxiety [38]. A former study with a large sample of Brazilian college students (845 women and 235 men) suggests that STAI-T results are closely related to mainly negative affect, which is an overlapping component of anxiety and depression [34]. If STAI-T scores are more reflective of depression than anxiety, this could explain their lack of association with cortisol levels.

STAI-S scores were also not correlated with cortisol levels. Another source of interference in the relationship between cortisol and anxiety resides in inter-individual differences in adaptation to stress [31]. In the context of academic stress, self-efficacy is one such individual factor. Academic self-efficacy refers to an individual’s conviction of being able to master tasks in educational settings, especially in exam situations [39, 40]. It is an important dispositional resource that attenuates threat appraisals and state anxiety [33, 41].

Self-efficacy can reduce anxiety in the context of an examination and also attenuate the negative effects that stress can have on academic performance [33, 35]. Self-efficacy may explain why, for our sample, neither cortisol nor anxiety levels were related to OSCE performance [6]. While high levels of stress and exam-related anxiety can impair working memory and the retrieval of learned information, with a negative effect on exam performance [42], self-efficacy has a protective effect and can intervene in this negative relationship. The results of our interviews support this interpretation: students reported that confidence had a calming effect and led to better self-perceived performance.

According to social cognitive theory, authentic success in dealing with a particular situation, realistic evaluative feedback, and physiological and psychological states are sources of information that help create student self-efficacy [43]. The OSCE can provide all these sources of information. In the interviews, students consistently referred to their previous OSCE experience to explain why they felt less anxious and more confident in their capabilities.

Our self-efficacy scores were positively and significantly correlated with exam performance, in line with many previous studies [44]. High self-efficacy is associated with better performance in clinical skills tests [45, 46]. This suggests that increasing levels of self-efficacy gave rise to progressively higher accomplishment [41]. This relationship was expected because competent performance requires not only knowledge and skills but also belief in one’s personal ability to use both effectively [45]. Thus, students who have adequate knowledge and skills but have low self-efficacy may show low performance.

Nevertheless, our results show that the relationship between self-efficacy and performance was only moderate. Figure 1 shows that some students’ self-efficacy over- or under-estimated actual performance. Poor self-efficacy beliefs can improve over the course of instruction, with students becoming more critical of their abilities if they receive accurate performance feedback. In line with the results of our interviews, previous research has shown that the OSCE offers valuable formative feedback whereby students learn which skills are important and need improvement [4, 5, 11–13, 47]. Feedback and success can improve self-efficacy, and students with a high sense of self-efficacy can learn to view a state of tension or anxiety as energizing in the face of a challenge [43]. Our interviews suggest that the relationship between OSCE-related anxiety and performance is moderated by self-efficacy (Fig. 2). This moderation effect should be investigated with appropriate statistical modeling in future studies.

## Conclusions

Our quantitative results indicate that a high proportion of our students reported stable and lingering negative affect. A significant proportion of students showed high state anxiety before the OSCE. However, neither stress nor anxiety was related to OSCE grades. Limitations in this study include the fact that there was only one sampling of cortisol 30 min before the OSCE. Collection of more saliva samples before, during and after the OSCE could possibly reveal further associations between cortisol levels, anxiety and performance. Qualitative analysis of our interviews, however, offers a reasonable explanation for the lack of association between cortisol, anxiety and OSCE scores in this sample of students. Students reported that previous experience with the OSCE allowed them to feel greater confidence in their capabilities. Student self-efficacy was correlated with their performance and may have protected them from the negative effects of stress and anxiety on performance. Overall, the results suggest that repeated exposure to clinical skills assessments followed by formative feedback may improve self-efficacy and moderate the negative effects of OSCE-related anxiety. Therefore, in spite of being stressful, the OSCE can provide meaningful learning experiences.

## Data Availability

The data analyzed during the current study are available from the corresponding author on request.

## Abbreviations

OSCE: Objective structured clinical examination
PT: Physical therapy
STAI: State-Trait Anxiety Inventory
STAI**-**S: State-Trait Anxiety Inventory - state anxiety
STAI**-**T: State-Trait Anxiety Inventory - trait anxiety
